# On the use of propensity scores in case of rare exposure

**DOI:** 10.1186/s12874-016-0135-1

**Published:** 2016-03-31

**Authors:** David Hajage, Florence Tubach, Philippe Gabriel Steg, Deepak L. Bhatt, Yann De Rycke

**Affiliations:** 1 APHP, Hôpital Louis Mourier, Département d’Epidémiologie et Recherche Clinique, 178 Rue des Renouillers, Colombes, 92700 France; 2 APHP, Hôpital Bichat, Département d’Epidémiologie et Recherche Clinique, 46 Rue Henri Huchard, Paris, F-75018 France; 3 APHP, Hôpital Bichat, Centre de Pharmacoépidémiologie (Cephepi), 46 Rue Henri Huchard, Paris, F-75018 France; 4 Univ Paris Diderot, Sorbonne Paris Cité, UMR 1123 ECEVE, Paris, F-75018 France; 5 INSERM, UMR 1123 ECEVE, Paris, F-75018 France; 6 INSERM, CIE-1425, Paris, F-75018 France; 7 FACT, DHU FIRE, Univ Paris-Diderot, Sorbonne Paris-Cité, Paris, F-75018 France; 8 LVTS, INSERM U-1148, Hôpital Bichat, HUPNVS, AP-HP, Paris, F-75018 France; 9 NHLI, Imperial College, Royal Brompton Hospital, London, UK; 10 Brigham and Women’s Hospital Heart & Vascular Center and Harvard Medical School, Boston, Massachusetts USA

**Keywords:** Propensity scores, Observational studies, Pharmacoepidemiology, Rare exposure, Hazard ratio, Monte Carlo simulations

## Abstract

**Background:**

Observational post-marketing assessment studies often involve evaluating the effect of a rare treatment on a time-to-event outcome, through the estimation of a marginal hazard ratio. Propensity score (PS) methods are the most used methods to estimate marginal effect of an exposure in observational studies. However there is paucity of data concerning their performance in a context of low prevalence of exposure.

**Methods:**

We conducted an extensive series of Monte Carlo simulations to examine the performance of the two preferred PS methods, known as PS-matching and PS-weighting to estimate marginal hazard ratios, through various scenarios.

**Results:**

We found that both PS-weighting and PS-matching could be biased when estimating the marginal effect of rare exposure. The less biased results were obtained with estimators of average treatment effect in the treated population (ATT), in comparison with estimators of average treatment effect in the overall population (ATE). Among ATT estimators, PS-weighting using ATT weights outperformed PS-matching. These results are illustrated using a real observational study.

**Conclusions:**

When clinical objectives are focused on the treated population, applied researchers are encouraged to estimate ATT with PS-weighting for studying the relative effect of a rare treatment on time-to-event outcomes.

**Electronic supplementary material:**

The online version of this article (doi:10.1186/s12874-016-0135-1) contains supplementary material, which is available to authorized users.

## Background

Post-marketing assessment of the risk and the benefit of a drug in real-world setting frequently relies on observational studies (such as prospective cohorts), comparing treated and untreated subjects on a time-to-event outcome. Effect of the drug exposure is then evaluated through the estimation of a hazard ratio [1–4].

By nature, observational studies may end up with an imbalance of baseline characteristics between exposed and unexposed subjects. If some of these characteristics are also associated with the outcome of interest, we are confronted with confounding factors, and the crude analysis of the treatment effect will be biased [5, 6].

Among the methods used to account for confounding factors in observational studies, propensity score (PS) analysis has been increasingly used [7]. PS analysis was developed to take into account the problem of confounding in observational studies [8], inducing baseline balance of measured confounding factors between groups of exposed and unexposed subjects. PS analysis works with two successive steps [9, 10]. The first step corresponds to the estimation of the probability of exposure conditional on baseline confounding factors. In the second step, these conditional probability estimates are used for the estimation of treatment effect. Several methods have previously been described and extensively compared [11–16]: adjustment on PS [8, 12], stratification on PS [11, 17], matching on PS [8, 14, 18], and PS-weighting estimation [15, 19]. Using empirical case studies and Monte Carlo simulations, several authors recently showed that PS-matching and PS-weighting more effectively reduced the imbalance between exposed and unexposed subjects in baseline covariates than the two other methods [11, 20], and should be the two preferred methods for the estimation of a marginal hazard ratio [16].

Unlike traditional regression analysis (i.e. incorporating exposure and confounding factors in the same regression model) which provides conditional estimation of the treatment effect, PS-weighting and PS-matching provide marginal estimation. While conditional effects denote an average effect for a specific strata defined by the vector of covariates included in the model, marginal effects denote an effect at the population level. The marginal estimation is similar to the causal estimation provided by a proper randomized clinical trial [10]. Furthermore, PS analysis outperforms conditional analysis when many confounding factors are taken into account: in this situation, conditional analysis may encounter convergence problems [21], particularly when the number of events of interest is small.

Several authors have discussed the use of PS analysis in some extreme situations such as small sample size [22] or rare outcome of interest [23–25]. But the use of PS analysis is also challenging in the case of rare exposure. This situation could frequently be encountered in pharmacoepidemiologic observational studies, particularly when study design does not require a high prevalence of exposure (for example, studies performed on electronic healthcare data, databases constituted with a nonspecific objective or analyzed for a different purpose than initially defined, evaluation of newly marketed drugs [26]). In this setting, the first step of PS analysis (i.e. conditional probability of treatment estimation) can be problematic, due to separation issues with the logistic model used for PS estimation, unless a large sample size is available. Although some recommendations encourage the use of alternative methods like disease risk score (DRS) in this setting [27, 28], to our knowledge, no study specifically assessed the effect of infrequent exposure on PS analysis. Even among the recent literature comparing DRS and PS based methods [29, 30], no article has explored the infrequent exposure setting.

Therefore, our objective was to evaluate the performance of PS-matching and PS-weighting to estimate the marginal hazard ratio associated with a rare exposure in the context of an observational study. An illustration is also provided from a real observational dataset, assessing the association between thiazolidinedione use and major cardiovascular outcomes.

## Methods

### A Monte Carlo simulation study

We used Monte Carlo simulations to examine the ability of some PS methods to estimate the marginal hazard ratio (HR) associated with a binary treatment in the context of rare exposure. They consisted in:

1. randomly generating independent datasets with several settings defined by exposure prevalence, covariates effect on exposure allocation and on outcome of interest, number of covariates, censoring rate, and exposure effect on outcome of interest (section ‘Data-generating process’);
2. applying each analytical method to analyze representative samples of each data set (section ‘Statistical analyses in simulated data sets’);
3. computing several criteria to evaluate and comparing the performance of each method (section ‘Performance criteria’).

### Definitions

In a cohort of *N* subjects, let *E* be an indicator variable denoting exposure status (*E*=1 for exposed subjects, *E*=0 otherwise), *Y* be an indicator variable of the event of interest (*Y*=1 if subject has experimented the event, *Y*=0 otherwise), and *t* the observed follow-up time. Let *B* and *C* be two baseline covariates, the first one being binary and the second one continuous. Finally, let *U* represent an unmeasured latent general health baseline variable.

### Data-generating process

We used a data-generating process derived from Havercroft et al., who provide an algorithm to generate data from a desired marginal structural model for survival outcome with time-dependent confounding on exposure causal effect [31]. In our simulation process, only baseline confounding was generated.

The key aspect of the algorithm proposed by Havercroft et al. is the use of an unmeasured uniformly distributed variable $U \sim \mathcal {U}(0,1)$ which represents a latent ‘general health’ process. A value of *U* close to 0 indicates poor health, and *U* close to 1 indicates good health.

First, for each subject, we randomly generate three normally distributed covariates (*X*_*B*_, *X*_*C*_, and *X*_*U*_) from the following multivariate normal distribution:

$$X = \left[X_{B}, X_{C}, X_{U}\right] \sim \mathcal{N}(0, \Sigma) $$

Variables *B*, *C* and *U* are then computed by applying the following transformations to *X*_*B*_, *X*_*C*_ and *X*_*U*_:

$$\begin{array}{*{20}l} &B = \left\{ \begin{array}{ll} 1 & \text{if}~X_{B} > 0 \\ 0 & \text{if}~X_{B} \leq 0 \\ \end{array} \right.,\\ &C = X_{C},\, \text{and}\\ &U \!= \!P(X_{U} \!\!<\!\! x) (\text{the cumulative distribution function of}\, X_{U}\!). \end{array} $$

By construction, *B* follows a Bernoulli distribution $\mathcal {B}(0.5)$, *C* follows a normal distribution $\mathcal {N}(0, \sigma _{C})$, and *U* follows a uniform distribution $\mathcal {U}(0, 1)$. *B*, *C*, *U* are related to each other through covariance parameters *σ*_*U*,*B*_, *σ*_*U*,*C*_ and *σ*_*B*,*C*_.

The exposure allocation *E* is drawn from a Bernoulli distribution $E \sim \mathcal {B}(p_{z})$, where

(1) $$ p_{z} = \text{logit}^{-1}\left(\delta_{0} + \delta_{B} B + \delta_{C} C\right).  $$

*δ*_0_ is the intercept, selected so that the prevalence of exposed subjects in the simulated sample is fixed at a desired proportion *p*, and *δ*_*B*_ and *δ*_*C*_ are the regression coefficients of this exposure allocation logistic model. For each targeted prevalence, we used an iterative process to determine the value of *δ*_0_ that induced the desired prevalence *p*:

1. We simulated 100,000 subjects, and computed the individual probabilities of exposure with Eq. 1. The average of these individual probabilities is the theoretical prevalence of exposure, *p* ^⋆^, in the sample.
2. Minimizing (*p* ^⋆^−*p*)^2^ (with the R function optim) allows us to obtain the parameter *δ*_0_ that induced desired prevalence of exposure *p*.
3. This process was repeated 1,000 times and values of *δ*_0_ were averaged to increase precision of the estimation.

An event time *T* with exponential distribution is generated from *U* as follows:

(2) $$ T = \frac{-log(U)}{\lambda \exp(\gamma E)},  $$

where *λ* is a constant baseline hazard function, and *γ* is the marginal effect of *E* on event time (i.e. *γ*=*l* *o* *g*(*H* *R*)). Censoring time *T*_*c*_ is drawn from a uniform distribution $\mathcal {U}(0,c)$ where *c* is chosen to achieve a desired censoring rate *r*_*c*_ in the simulated sample. Finally, the observed time-to-event outcome is obtained with the following decision rule:

$$Y = 1, t = T~\text{if}~T \leq T_{c} $$

$$Y = 0, t = T_{c}~\text{if}~T > T_{c} $$

**Fig. 1:**
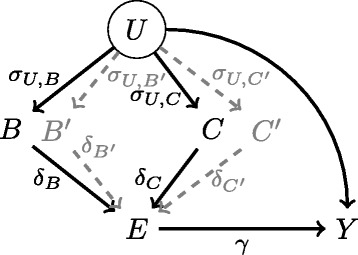
Directed acyclic graph corresponding to the data-generating algorithm

Applied for *N* subjects, this algorithm generates a sample corresponding to the directed acyclic graph represented on Fig. 1. The key mechanism by which the algorithm generates confounding in the estimation of the marginal exposure effect is the way in which the exposure *E* and the time *t* to event outcome *Y* depends (directly or undirectly) both on *U*. The relationship between *U* and *Y* is straightforward, as *U* is used to generate event times *T* (Eq. 2). The relationship between *U* and *E* is mediated by the two other covariates *B* and *C*, which are ‘natively’ correlated with *U* (through parameters *σ*_*U*,*B*_ and *σ*_*U*,*C*_), and then used to calculate the probability of exposure allocation (Eq. 1). There is confounding due to *U* being a common ancestor of *E* and *Y*. *B* and *C* are sufficient to adjust for confounding, because *E* is independent of *U* given *B* and *C*.

In all simulations, the following parameters were fixed:

- *N*=10,000
- *λ*=0.1
- ${\sigma _{U}^{2}} = {\sigma _{B}^{2}} = {\sigma _{C}^{2}} = 1$

We allowed the following parameters to vary across simulations:

- the prevalence of exposure: *p*∈{1 *%*,2 *%*,5 *%*,10 *%*};
- the strength of the correlation between covariates *B* and *C*: *σ*_*B*,*C*_∈{0,0.1,0.3,0.5} (no, weak, moderate, or strong correlation);
- the strength of the association between covariates and *U*: *σ*_*U*,*B*_=*σ*_*U*,*C*_∈{0,0.1,0.3,0.5} (no, weak, moderate, or strong association);
- the strength of the association between covariates and exposure allocation: $\exp (\delta _{B}) = \exp (\delta _{C}) \in \{1, 1.2, 1.5, 2\}$ (no, weak, moderate, or strong association);
- the strength of the marginal association between exposure and outcome: $HR = \exp (\gamma) \in \{1, 1.2, 1.5, 2\}$ (no, weak, moderate, or strong association);
- the censoring rate: *r* *c*∈{20 *%*,50 *%*,80 *%*};

For the intelligibility of the description of the data-generating process, only two covariates (*B* and *C*) were previously described. In order to study the impact of the number of confounding factors, two additional covariates, $B^{\prime }$ and $C^{\prime }$, were generated in some scenarios, according to the same process. In these scenarios, $B^{\prime }$ is binary, $C^{\prime }$ is continuous, and *B*, $B^{\prime }$, *C*, $C^{\prime }$, and *U* are related to each other through covariance parameters $\sigma _{U,B} = \sigma _{U,B^{\prime }}$, $\sigma _{U,C} = \sigma _{U,C^{\prime }}$, $\sigma _{B,C} = \sigma _{B^{\prime },C^{\prime }}$ and $\sigma _{B,B^{\prime }} = \sigma _{C,C^{\prime }} = 0$. These two additional covariates are represented in gray on Fig. 1. A detailed document that encapsulates the data-generating process and all of the simulation scenarios in one place is included in the supplemental material (Additional file 1).

### Statistical analyses in simulated data sets

First, in each simulated cohort, random representative samples of increasing size were selected. When studying a rare exposure and limited sample sizes, it is not uncommon to have no event *Y* in the exposed group. These samples could not be analysed. Dropping all samples with no events in the exposed group would lead to over-represent samples with enough events, and would therefore break the simulation settings when studying small sample sizes. To prevent this situation, samples were not selected according to fixed sample sizes, but according to fixed numbers of events *y* in the exposed group. More precisely, in each simulated cohort, we selected the first set of subjects among which there were *y* events in the exposed group, with *y* varying from 2 to 200, with increment of 2. This allows having enough events in all analysed samples, while ensuring the selection of representative samples of the underlying cohort.

Then, each representative sample was analyzed with the following statistical methods.

#### Propensity score (PS) analysis with PS-weighting

First, individual PS (i.e. individual probability of being exposed given baseline covariates) was estimated with the following logistic model:

(3) $$ PS = \text{logit}^{-1}\left(\hat{\delta}_{0} + \hat{\delta}_{B} B + \hat{\delta}_{C} C\right)  $$

The propensity score of each patient was estimated from the predicted probability of treatment given his(her) covariates.

Then, we applied the Cox proportional hazards model given by the following equation:

(4) $$ \lambda(t) = \lambda_{0}(t)\exp(\hat{\gamma} E)  $$

with each subject weighted using the propensity score, and robust standard error estimator [32].

The PS related literature differentiates between the average treatment effect in the entire eligible population (ATE) and the average treatment effect in treated subjects (ATT) [33]. Indeed, two types of weights could be used depending on the desired estimate, as follow:

$$W_{ATE} = \frac{E}{PS} + \frac{1-E}{1-PS} $$

$$W_{ATT} = E + \frac{PS(1-E)}{1-PS} $$

With ATE weights, we considered stabilized weights [34, 35] by multiplying previous (un-stabilized) weights by $E\bar {p} + (1-E)(1-\bar {p})$ (where $\bar {p}$ is the overall probability of being exposed, i.e. the prevalence of exposure estimated in the selected sample).

#### Propensity score (PS) analysis with PS-matching

First, individual PS were estimated with Eq. 3. Then, we used greedy nearest-neighbour 1:1 matching within specified caliper widths to form pairs of exposed and unexposed subjects matched on the logit of the propensity score, without replacement. We used calipers of width equal to 0.2 of the standard deviation of the logit of the propensity score as this caliper width has been found to perform well in a wide variety of settings [36].

Once matching was completed, we used an univariate Cox proportional hazards regression model with exposure as the only variable to estimate ATT. We used robust estimate of the standard error of the regression coefficient that accounted for the clustering within matched sets [32].

### Performance criteria

We performed 5000 simulations per scenario. Results were assessed in terms of the following:

- Bias of the exposure effect estimation: $E(\hat {\gamma } - \gamma)$.
- Root mean squared error (RMSE) of the exposure effect estimation, defined as: $\sqrt {E((\hat {\gamma } - \gamma)^{2})}$.
- Variability ratio of the exposure effect, defined as: $\frac {\frac {1}{5000}\sum _{i=1}^{5000}\hat {SE}(\hat {\gamma }_{i})} {\sqrt {\frac {1}{4999}\sum _{i=1}^{5000}\left (\hat {\gamma }_{i}-\bar {\hat {\gamma }}\right)^{2}}}$, where $\hat {SE}(\hat {\gamma }_{i})$ is the estimated standard error of exposure effect $\hat {\gamma }$ in the simulation *i*. A ratio >1 (or <1) suggests that standard errors overestimate (or underestimate) the variability of the estimate of exposure effect [25, 37].
- Coverage: proportion of times *γ* is enclosed in the 95 % confidence interval of *γ* estimated from the model.

The mean sample size *n* were also computed for each scenario.

The data-generating algorithm used in this simulation study allows to generate data with a desired level of ATE. But PS-matching and PS-weighting using ATT weights methods do not provide the same type of estimation (ATT). For these two methods in each evaluated scenario, performance metrics were estimated relative to the corresponding theoretical ATT hazard ratios.

In case of null treatment effect, the true marginal effect is null and do not vary over the sample. Theoretical ATE and ATT are equal: $HR = \exp (\gamma) = 1$. In case of non-null treatment effect, theoretical ATT were computed as followed:

- Using the parameters of the select scenario, we simulated a cohort of 100,000 subjects. Whatever the ‘real’ exposure status simulated, we generated two potential event times for each subject: first assuming that the subject was unexposed and then assuming that the subject was exposed to the treatment.
- In the sample regrouping each subject twice (once with the outcome under treatment, and once with the outcome with no treatment), we fitted a Cox model using only subjects who were “really" exposed. The obtained coefficient corresponded to the ATT of the population.
- We repeated this process 1,000 times and averaged the values to increase the precision of this estimation.

### Software

All simulations and analyses were performed using R software version 3.1.1 (R Foundation for Statistical Computing, Vienna, Austria). Critical parts (in terms of performances, mostly data sets generation procedure) of the simulation program were coded using C++, and integrated into R code with the help of Rcpp package [38].

## Results

Results were displayed using a reference configuration: prevalence of exposure *p*= 5 %, moderate association between confounding factors and outcome (*σ*_*U*,*B*_=*σ*_*U*,*C*_=0.3), moderate association between confounding factors and exposure ($\exp (\delta _{B}) = \exp (\delta _{C}) = 1.5$), no marginal association between exposure and outcome ($\exp (\gamma) = HR = 1$), two independant confounding factors (one binary, one continuous, *σ*_*B*,*C*_=0), and a censorting rate *r*_*c*_ of 50 %. Then, the effects of change of each of the simulation parameters (compared to the value used in the reference configuration) were reported. More precisely, when the value of a parameter is changed, all other parameters are fixed to the value used in the reference configuration.

The strength of confounding was defined in four classes:

- No confounding: *σ*_*U*,*B*_=*σ*_*U*,*C*_=0 and $\exp (\delta _{B}) = \exp (\delta _{C}) = 1$
- Weak confounding: *σ*_*U*,*B*_=*σ*_*U*,*C*_=0.1 and $\exp (\delta _{B}) = \exp (\delta _{C}) = 1.2$
- Moderate confounding: *σ*_*U*,*B*_=*σ*_*U*,*C*_=0.3 and $\exp (\delta _{B}) = \exp (\delta _{C}) = 1.5$
- Strong confounding: *σ*_*U*,*B*_=*σ*_*U*,*C*_=0.5 and $\exp (\delta _{B}) = \exp (\delta _{C}) = 2$

To make the comparison across the different scenarios possible, table and figures of this section report the mean sample size *n*.

### Results for the reference configuration

Results for the reference configuration previously defined are presented in Table 1.

**Table 1:** Results for the reference configuration

Method	*y*	*n*	Bias	V ratio	RMSE	1-coverage	% match
PSW-ATE	10	364	0.056	0.914	0.406	0.091	
	20	728	0.028	0.982	0.271	0.065	
	30	1092	0.018	1.009	0.216	0.057	
PSW-ATT	10	364	0.026	0.983	0.321	0.060	
	20	728	0.013	1.019	0.222	0.047	
	30	1092	0.008	1.031	0.180	0.046	
PS-matching	10	364	0.051	0.925	0.473	0.062	99.0
	20	728	0.026	0.964	0.316	0.056	99.5
	30	1092	0.017	0.990	0.250	0.053	99.7

Bias, variability ratio, RMSE, and 1-coverage according to analytical method, number of events in the exposed group *y*, and mean analyzed sample size *n*, for one scenario (*p*=5 *%*, *σ*_*U*,*B*_=*σ*_*U*,*C*_=0.3, *σ*_*B*,*C*_=0, $\exp (\delta _{B}) = \exp (\delta _{C}) = 1.5$, *H* *R*=1, 2 confounding factors, censoring rate *r*_*c*_=50 *%*). The mean percentage of matched exposed subjects is reported for the PS-matching method

When *y*=20 (20 events in the exposed group, approximatively 700 analyzed subjects overall), PS-weighting using ATE weights (PSW-ATE) and PS-matching were the most biased methods, followed by PS-weighting using ATT weights (PSW-ATT), and the latter was the only method having coverage below the nominal level. Bias and coverage deteriorated when sample size decreased (*y*=10, approximatively 350 analyzed subjects overall), particularly for PSW-ATE. When sample size increased (*y*=30, approximatively 1100 subjects overall), PSW-ATE and PS-matching showed very similar results, and PSW-ATT was still the best method according to bias and coverage performance parameters.

Variability ratios suggested that standard errors underestimate the variability of the exposure effect estimate for methods PSW-ATE and PS-matching when the sample size was low. Variability ratios increased with the sample size, and became clearly larger than 1 for PSW-ATT method (meaning that standard errors tend to be overestimated). The lowest RMSE were observed with the PSW-ATT method.

Table 2 reports the distribution of ATE and ATT weights according to exposure status. Despite the use of stabilized weights, ATE (but not ATT) weights could reach extreme values in the exposed population.

**Table 2:** Distribution of ATE and ATT weights for the reference configuration

		ATE				ATT			
		Weights				Weights			
*y*	E	Mean	Var	Min	Max	Mean	Var	Min	Max
10	0	1.000	0.001	0.887	3.596	0.052	0.001	0.000	2.940
	1	0.995	0.383	0.064	17.072	1.000	0.000	1.000	1.000
20	0	1.000	0.001	0.922	2.305	0.052	0.001	0.000	1.436
	1	0.999	0.296	0.064	10.461	1.000	0.000	1.000	1.000
30	0	1.000	0.001	0.932	1.727	0.052	0.001	0.001	0.848
	1	0.999	0.265	0.109	10.465	1.000	0.000	1.000	1.000

Mean, variance, minimum and maximum ATE and ATT weights according to type of weights, number of events in the exposed group *y* and exposure status *E* for one scenario (*p*=5 *%*, *σ*_*U*,*B*_=*σ*_*U*,*C*_=0.3, *σ*_*B*,*C*_=0, $\exp (\delta _{B}) = \exp (\delta _{C}) = 1.5$, *H* *R*=1, 2 confounding factors, censoring rate *r*_*c*_=50 *%*)

### Effect of the prevalence of exposure

**Fig. 2:**
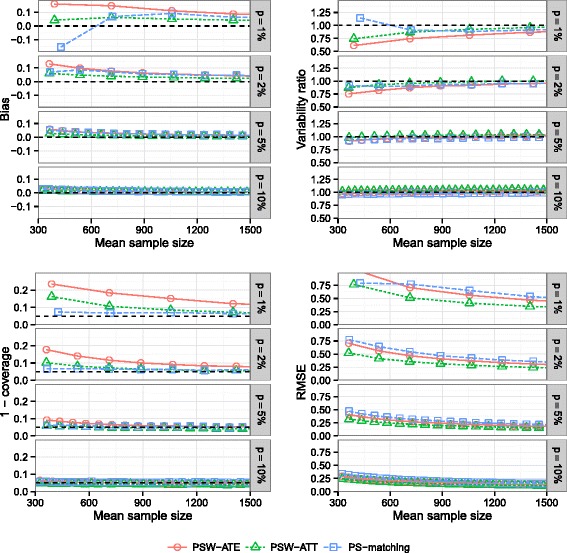
Effect of exposure prevalence. Bias of exposure effect, variability ratio, 1 - coverage and RMSE according to *prevalence* *p* of exposure and mean sample size, for one continuous and one dichotomous confounder, *σ*_*U*,*B*_=*σ*_*U*,*C*_=0.3, *σ*_*B*,*C*_=0, $\exp (\delta _{B}) = \exp (\delta _{C}) = 1.5$, *r*_*c*_=50 *%* and *H* *R*=1, with weighting by inverse of PS using ATE and ATT weights and PS-matching

Figure 2 show that bias decreased when sample size and/or prevalence increased. Bias decreased more slowly for PSW-ATE than for PSW-ATT. At lower prevalences of exposure (1 and 2 %), PS-matching encountered severe convergence issues, which explained the appearance of the corresponding bias curve. At this level of prevalence, neither PSW-ATE nor PSW-ATT had satisfactory coverage properties unless a large sample size was analyzed (Fig. 2), the worst method being the use of ATE weights. Standard errors were underestimated at lower levels of prevalence and/or sample size, and became slightly overestimated for PSW-ATT method when prevalence and sample size increased. PSW-ATT method had the lowest RMSE levels. When prevalence was 10 %, bias, coverage and variability ratio were satisfactory for all methods.

### Effect of the marginal effect of exposure on outcome event

**Fig. 3:**
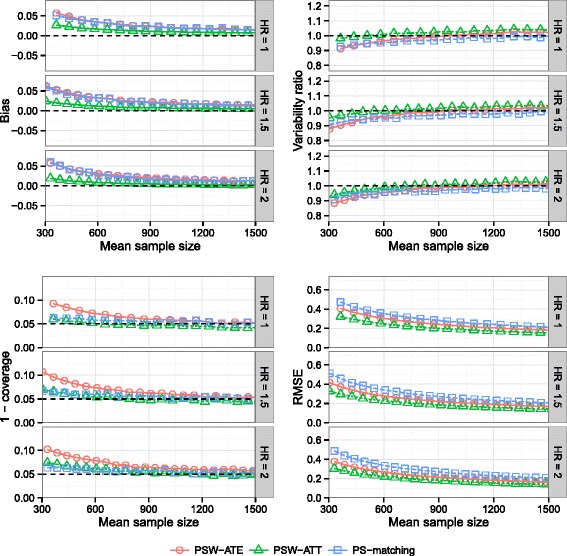
Effect of theoretical hazard ratio. Bias of exposure effect, variability ratio, 1 - coverage and RMSEw according to *theoretical exposure effect* (HR) and mean sample size, for one continuous and one dichotomous confounder, *σ*_*U*,*B*_=*σ*_*U*,*C*_=0.3, *σ*_*B*,*C*_=0, $\exp (\delta _{B}) = \exp (\delta _{C}) = 1.5$, *r*_*c*_=50 *%* and *p*=5 *%*, with weighting by inverse of PS using ATE and ATT weights and PS-matching

Influence of theoretical HR is illustrated on Fig. 3. In these scenarios, theoretical values of ATT hazard ratio (used to evaluate the performance of PS-matching and PSW-ATT methods) were 1, 1.471 and 1.935, for theoretical values of ATE hazard ratio of 1, 1.5 and 2 respectively.

All results were mostly unchanged with varying effect of exposure. PSW-ATT was both the less biased method and had the lowest RMSE levels.

### Effect of the strength of confounding

**Fig. 4:**
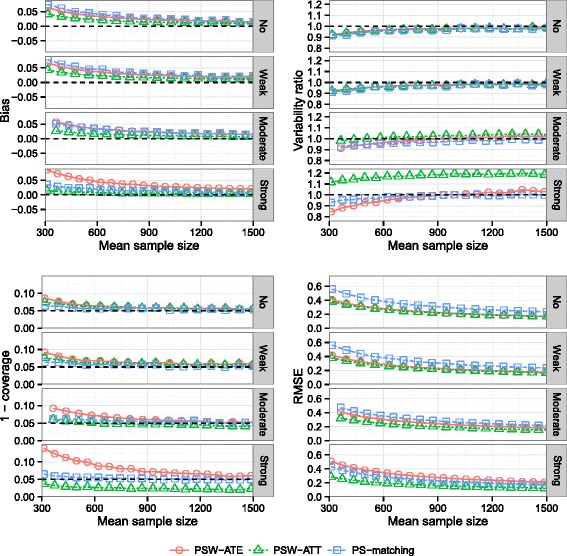
Effect of strength of confounding. Bias of exposure effect, variability ratio, 1 - coverage and RMSE according to *strength of confounding* and mean sample size, for one continuous and one dichotomous confounder, *σ*_*B*,*C*_=0, *H* *R*=1, *r*_*c*_=50 *%* and *p*=5 *%*, with weighting by inverse of PS using ATE and ATT weights and PS-matching

Results are illustrated on Fig. 4. In terms of bias, increasing the strength of confounding had a favorable impact on PSW-ATT and PS-matching methods. In contrast, with PSW-ATE method, bias increased with the strength of confounding.

At strong level of confounding, standard errors were overestimated when using PSW-ATT. Consequently, coverage probabilities were greater than the nominal coverage probability, but PSW-ATT remained the most performant method in terms of RMSE.

### Effect of the number of confounding factors

**Fig. 5:**
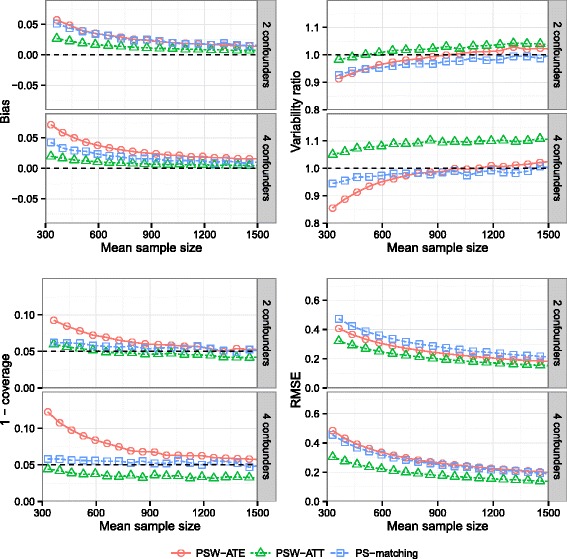
Effect of the number of confounders. Bias of exposure effect, variability ratio, 1 - coverage and RMSE according to *number of confounders* (2 or 4 confounders) and mean sample size, for *σ*_*U*,*B*_=*σ*_*U*,*C*_=0.3, *σ*_*B*,*C*_=0, $\exp (\delta _{B}) = \exp (\delta _{C}) = 1.5$, *H* *R*=1, *r*_*c*_=50 *%* and *p*=5 *%*, with weighting by inverse of PS using ATE and ATT weights and PS-matching

Results are illustrated on Fig. 5. The number of confounding factors had a important impact on the bias found with PSW-ATE method, in contrast to the one found with methods estimating ATT. Increasing the number of confounders increased the variability ratio of PSW-ATT method, which consequently seemed too conservative. Conversely, coverage properties of PSW-ATE method deteriorated with the transition from two to four confounders. Again, the method with the lowest RMSE values was PSW-ATT, whatever the number of confounding factors.

### Effect of the censoring rate

**Fig. 6:**
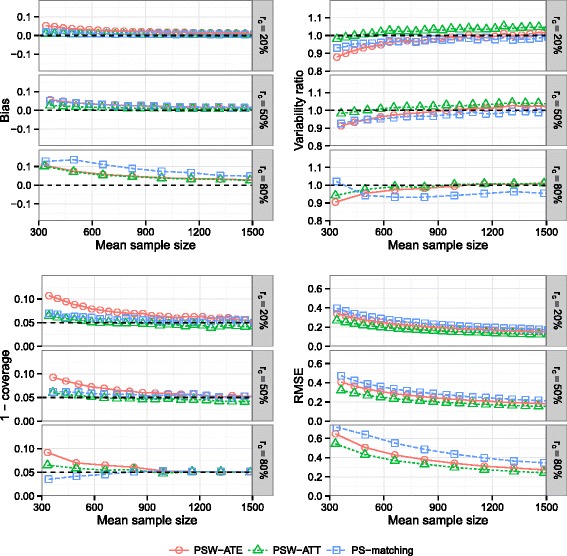
Effect of censoring rate. Bias of exposure effect, variability ratio, 1 - coverage and RMSE according to *censoring rate* (*r*_*c*_) and mean sample size, for one continuous and one dichotomous confounder, *σ*_*U*,*B*_=*σ*_*U*,*C*_=0.3, *σ*_*B*,*C*_=0, $\exp (\delta _{B}) = \exp (\delta _{C}) = 1.5$, *H* *R*=1 and *p*=5 *%*, with weighting by inverse of PS using ATE and ATT weights and PS-matching

Results are illustrated on Fig. 6. Bias increased with increasing censoring rate for all methods. At the lower level of censoring (*r*_*c*_=20 *%*), PSW-matching method was less biased than PSW-ATE method. The opposite was observed at the highest level of censoring. Bias found with PSW-ATT method never exceeded the bias found with PSW-ATE method.

Again, coverage properties and RMSE levels were more satisfactory with PSW-ATT than with PSW-ATE method.

### Effect of the correlation between covariates *B* and *C*

**Fig. 7:**
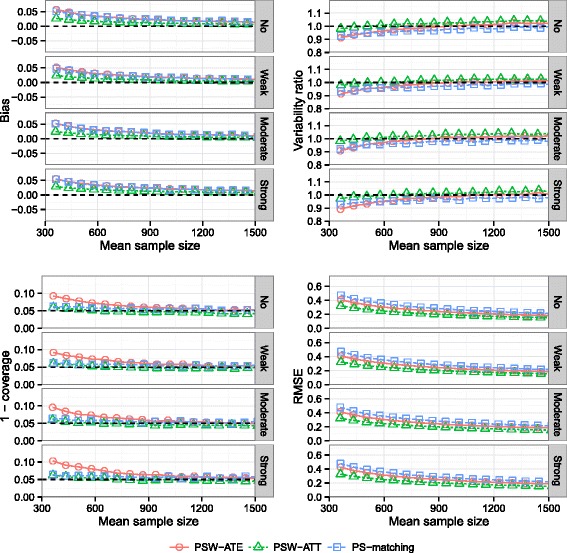
Effect of correlation between covariates. Bias of exposure effect, variability ratio, 1 - coverage and RMSE according to *correlation between covariates B and C* (*σ*_*B*,*C*_) and mean sample size, for one continuous and one dichotomous confounder, *σ*_*U*,*B*_=*σ*_*U*,*C*_=0.3, $\exp (\delta _{B}) = \exp (\delta _{C}) = 1.5$, *H* *R*=1 and *p*=5 *%*, with weighting by inverse of PS using ATE and ATT weights and PS-matching

Results are illustrated on Fig. 7. Whatever the method, the overall effect of the correlation level between confounding factors was modest.

### Real observational dataset illustration

To illustrate these results, we applied the PS methods described above in an already published real observational study [39]. The objective of this study was to compare the occurrence of death, non-fatal myocardial infaction, and congestive heart failure in patients with diabetes, according to the use of thiazolidinedione (TZD), in the REACH (REduction of Atherothrombosis for Continued Health) Registry, an international prospective cohort of patients with either established atherosclerotic arterial disease or at risk for atherothrombosis [40–43]. Patients were enrolled in 44 countries between December 2003 and December 2004. In each country, the protocol was submitted to the institutional review boards according to local requirements, and signed informed consent was obtained for all patients.

From the REACH Registry, we selected 28,332 patients with type 2 diabetes and available data on TZD use. This population (mean age 68 years, standard deviation 9.6 years, 61 % of male) has been previously described, and is composed of 4997 TZD users at baseline (prevalence of exposure 17 %).

**Fig. 8:**
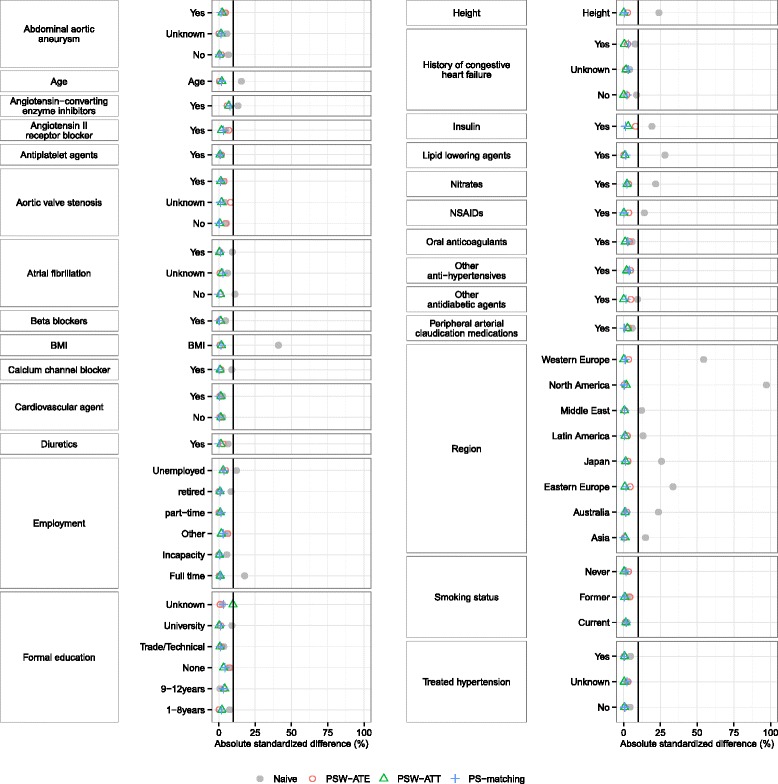
Imbalances in the REACH cohort, defined as the standardized means differences of covariate values between the two treatment groups. Solid black line represents an absolute standardized difference of 10 %

The list of co-variables used to calculate the propensity score was the same as in the original publication, and included age, geographic region of enrolment, height, body mass index, smoking status, atrial fibrillation/flutter, history of congestive heart failure, treated hypertension, use of lipid-lowering agents, anti-platelet agents, oral anti-coagulants, non-steroidal anti-inflammatory agents, diuretics, cardiovascular agents, peripheral arterial claudication medications, insulin, and use of other anti-diabetic agents. Before the use of PS methods, some known risk factors of cardiovascular events were imbalanced between TZD users and non-users, according to their absolute standardized differences (ASD) (Fig. 8). Compared to the ASD observed in the previous simulations (data not shown), some variables had ASD comparable to the ‘weak’ confounding condition (like continuous ‘age’ or binary ‘Atrial fibrillation’ variables), but also comparable to the ‘moderate’ (like continuous ‘BMI’ or binary ‘Insulin’ variables), or ‘strong’ confounding condition (like the multimodal ‘region’ variable). After application of the estimated propensity score to the entire dataset, all variables including those not used in the PS estimation (like formal education and employment) were correctly balanced between TZD users and non-users.

In this application, all event types where regrouped into the same composite outcome (time to the occurrence of the first event). An event occurred in 12 % of subjects. TZD effect was estimated with PS-matching and PS weighting approaches. None of these methods found a significant effect of TZD. No treatment effect heterogeneity was detected (test for homogeneity of the TZD effect across deciles of the PS, *p*-value = 0.5425).

**Fig. 9:**
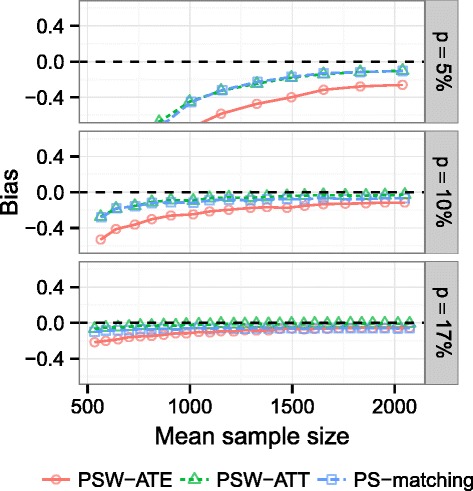
Real observational dataset illustration. Bias of TZD effect estimation in the REACH cohort, using PS-matching and PS-weighting approaches, according to prevalence *p* and mean sample size

We then 1) randomly dropped some TZD users to create a new dataset with a pre-specified lower prevalence of exposure 2) applied the three PS-based methods to a representative sample of this new dataset. This two-step process was repeated 2,000 times for prevalences ranging from 17 % (real) down to 5 % and increasing sample sizes (selected according to the number of events in the exposed group, like in our simulations). We chose to limit the exploration of the real observational dataset to prevalence of exposure higher than 5 %, because event rate was only 12 % in the REACH cohort, and the number of events in the exposed group is then limited. Bias (relatively to the TZD effect estimated by each method applied in the entire cohort) was averaged and drawn on Fig. 9.

As demonstrated in the simulation study, we observed that ATE estimations were severely biased compared to TZD effect estimated in the full dataset, particularly for the smallest prevalences, even if a large sample size was analyzed. In contrast, ATT estimations through PS-weighting using ATT weights were uniformly less biased, whatever the prevalence and the sample size used. In this application, results observed with PS-matching and PSW-ATT methods seemed superimposed, but this is due to the extremely poor performances of PSW-ATE method, and bias was actually higher with PS-matching than with PSW-ATT.

## Discussion

The present simulation study shows that in case of rare exposure, PS-weighting or PS-matching can be biased for estimating the marginal hazard ratio of an exposure. This result was particularly clearcut with PS-weighting analysis using ATE weights, even if stabilized weights were used across all analyses. All methods were converging to their theoretical value with increasing sample size and/or prevalence, but the use of ATE weights and PS-matching needed more subjects than the use of ATT weights. This result leads to limiting the use of PS analysis in case of rare exposure if a sufficient number of subjects is not available, and to favour PS-weighting method using ATT weights when the number of subjects is limited.

Nevertheless, ATT estimation is not consistent with the study objectives in all cases. Small prevalence of exposure could be encountered in two main situations. First, a drug on the market for a long time, and actually little prescribed: in this situation, estimating ATE may not be of great interest, and estimating ATT makes more clinical sense. Second, a newly marketed drug, that is not intended to remain uncommon: this situation is a subject of special attention from the health authorities, and early assessment of the drug effect if the entire population was exposed would be of great interest to public health policy. Our simulation results stress the importance of looking for methods less influenced by exposure prevalence.

The concerns with ATE estimation in case of rare exposure were sustained by our real dataset illustration. The number of potential confounders taken into account were high, and some variables had absolute standardized differences comparable to the ‘moderate’ and ‘strong’ confounding conditions of the simulations. We assumed from the former simulation results that the high degree of bias observed with PSW-ATE method in the REACH study is due to the strength of confounding and the number of confounders present in the database, which had a large impact on ATE estimates. Hence, results observed in the REACH study were consistent with the simulation results.

Pirracchio et al. [22] concluded from their simulation study that ‘even in case of small study samples or low prevalence of treatment, both propensity score matching and inverse probability of treatment weighting can yield unbiased estimations of treatment effect’. However this study explored more specifically the context of small sample size (ranging from 1000 down to 40) rather than low prevalence of exposure (ranging from 50 % down to 20 %). While some conventions exist on the definition of a rare disease [44], there is, to our knowledge, no such definition of a rare exposure. Nevertheless, we felt that a 1:4 exposure ratio represented a quite common exposure, and more extreme situations could be encountered in observational studies, for example those focusing on a newly marketed medications or when many therapeutic strategies are available. To the best of our knowledge, the present study is the first to focus on the performance of PS-based methods in the context of a rare exposure (10 % down to 1 %) and small sample sizes. This explains that, unlike Pirracchio et al., we conclude that PS-based methods could lead to rather biased estimates when prevalence is low, particularly when estimating average treatment effect in the whole population.

Without focusing specifically on rare exposure issue, Austin et al. have compared the performance of different propensity score methods for estimating absolute effects [45] and relative effects [16] of treatments on survival outcomes. In these two simulation studies, low prevalences of exposure were also simulated. The authors did not observe any major performance issue using PS-weighting or PS-matching when proportion of treated subjects was fixed to 10 % or 5 %. For the estimation of absolute effects, they reported that PS-matching tended to decrease bias compared with PS-weighting approaches. However, all methods compared in this article were applied on simulated cohorts of 10,000 subjects. With fewer subjects, we observed that 1) all methods could be biased, 2) PS-weighting using ATT weights outperformed PS-matching for the estimation of ATT, and 3) PS-weighting using ATE weights was the method which performance deteriorates most with the decrease of exposure prevalence.

The context of rare exposure is also addressed by authors interested in ‘the prognostic analogue of the propensity score’, a.k.a. disease risk score (DRS) [29, 46, 47]. Actually, Effective Health Care Program recommends the use of disease risk score instead of propensity score when the exposure is infrequent [27, 28], but without defining when an exposure should be considered as infrequent. No study has compared propensity and disease risk score methods for the estimation of an exposure effect in the context of rare exposure. Arbogast et al. [29] compared the performance of disease risk score, propensity score and traditional multivariable regression to evaluate a treatment effect on a Poisson outcome, but prevalence of exposure was fixed to 10 %, and computations were based on the analysis of samples consisting of 10,000 subjects. The authors concluded that all methods performed well when there was an adequate number of events per covariates. Our simulation results also suggest that all PS-based methods are unbiased at this level of prevalence when a large sample size is analyzed. Wyss and colleagues [30] compared PS and DRS matching, and concluded that the use of DRS yielded to match more exposed subjects than the use of PS, and this improved the precision of the effect estimate. However, the prevalence of exposure was fixed to 30 % in all the scenarios considered. Intuitively, this advantage of DRS should be less apparent in case of lower prevalence of exposure. Among the scenarios and sample sizes explored in the present article, the percentages of matched exposed subjects were high (Q25 = 99.7 %, Q50 = 99.8 %, Q75 = 99.9 %). Thus, further investigation is needed to assess if DRS really performs better than PS in the context of rare exposure, especially as the relative performance of the different DRS-based methods for estimating ATE and ATT are today a research area [27].

In the setting of rare exposure, we found that application of PS-based methods could provide biased estimates unless a large sample size was available. PS method being a two-step estimator, the appropriateness of the estimation in the second step relies on correct modelling of the probability of exposure during the first step, which could be problematic in case of infrequent exposure, due to separation issues. Of note, alternative strategies than logistic regression have been proposed to estimate individual probability of exposure [48], but we found no information about how they would be affected by a rare exposure issue.

All PS methods rely on the validity of estimates of individual exposure probability, and thus on the validity of the logistic regression fitted for these estimations. A classical rule when fitting a logistic model is to have an adequate number of outcomes per predictor (at least five or ten outcomes per predictor [49, 50]). This explains why we chose to limit the number of confounding factors in our simulations: in case of small prevalence of exposure, the number of exposed subjects, and therefore the number of variables that could be included in the logistic model, is limited. The bias observed in some of our simulations could not be explained by an inadequate number of exposed subjects per co-variables in all cases: even with only two confounding factors, bias was still present with a sample size of 500 subjects and an exposure prevalence of 5 % (and thus 25 exposed subjects on average) or 10 % (50 exposed subjects on average). Therefore, the previously mentioned ‘rule of thumb’ fails to provide sufficiently accurate estimates of individual exposure probability, particularly when estimating ATE with PS-weighting method.

Other reasons might explain that the ATT estimates were more reliable that ATE estimates in the context of rare exposure. First, ATT estimates apply to a much more homogeneous population, so less confounding might be involved. Another reason might be that strong confounding and limited overlap between treatment groups leads to a violation of the positivity assumption. We observed that ATE (but not ATT) weighting can yield extreme weights in the exposed population, as well as biased and highly variable estimates.

One of the strengths of this study is the use of an algorithm which directly generates data with desired marginal HR and confounding on exposure causal effect. Indeed, several simulations studies evaluating the performance of PS methods to estimate marginal HR used a conditional model to link the outcome with the exposure and (time-dependent or not) confounding factors, even though the measures used to estimate exposure effect on outcome are sometimes non-collapsible [51, 52] (i.e. conditional and marginal treatment effects will not coincide). Two more approximate strategies are typically used to deal with this issue: the use of a high number of simulations to determine the value of the conditional hazard ratio that induced the desired marginal hazard ratio [16]; or the *post-hoc* verification that conditional and marginal treatment effects are in the same range [53]. Another solution is to use a collapsible estimate of exposure effect, like risk differences [15], but this type of estimator is less used to report the effect of an exposure in real studies. Nevertheless, even if we did not use a conditional model to generate simulated datasets, a rather similar issue remains in this article: our algorithm simulates a desired hazard ratio in the entire cohort (ATE), but not a desired hazard ratio in the treated population (ATT). Thus, a possible explanation for the discrepancies between methods estimating ATE and ATT is that they are compared to different theoretical values of the treatment effect. However, this issue was minimized in this study 1) by choosing a null treatment effect in the majority of the reported scenarios (in this case, ATE and ATT are both null), and 2) by estimating the theoretical ATT as precisely as possible with a large number of simulations of potential outcomes in other cases. Moreover, if this estimation of theoretical ATT was not sufficiently accurate, this would probably disadvantage methods estimating ATT, which reinforce the findings of this study.

## Conclusions

In conclusion, this simulation study showed that in case of rare exposure, marginal treatment effect estimation through propensity score analysis can be severely biased, in particular when focusing on average treatment effect in the entire eligible population (ATE). When clinical objectives are focused on the treated population, PS-weighting using ATT weights should be the preferred estimator of the treatment effect. Further work in this area is needed to provide improved analytical strategies for the estimation of the marginal treatment effect in the context of an observational study with a rare exposure.

## Availability of data and materials

The R code corresponding to the data-generating process and the statistical methods used in this article can be obtained on request to David Hajage (david.hajage@aphp.fr).

Real dataset supporting the findings (REACH Registry) can be obtained on request to Philippe Gabriel Steg (gabriel.steg@aphp.fr).

## Additional file

Additional file 1 Data-generation process and simulated scenarios. (DOCX 119 kb)

## Abbreviations

ATE: average treatment effect
ATT: average treatment effect in the treated
DRS: disease risk score
HR: hazard ratio
PS: propensity score
PSW: propensity score weighting
REACH: REduction of Atherothrombosis for Continued Health
TZD: thiazolidinedione

## References

[1] Rafaniello C, Lombardo F, Ferrajolo C, Sportiello L, Parretta E, Formica R, Potenza S, Rinaldi B, Irpino A, Raschetti R, Vanacore N, Rossi F, Capuano A (2014). Predictors of mortality in atypical antipsychotic-treated community-dwelling elderly patients with behavioural and psychological symptoms of dementia: a prospective population-based cohort study from Italy. Eur J Clin Pharmacol.

[2] Weinhandl ED, Gilbertson DT, Collins AJ, Foley RN (2014). Relative safety of peginesatide and epoetin alfa. Pharmacoepidemiol Drug Saf.

[3] Eftekhari K, Ghodasra DH, Haynes K, Chen J, Kempen JH, VanderBeek BL (2014). Risk of retinal tear or detachment with oral fluoroquinolone use: a cohort study. Pharmacoepidemiol Drug Saf.

[4] Beigel F, Steinborn A, Schnitzler F, Tillack C, Breiteneicher S, John JM, Van Steen K, Laubender RP, Göke B, Seiderer J, Brand S, Ochsenkühn T (2014). Risk of malignancies in patients with inflammatory bowel disease treated with thiopurines or anti-TNF alpha antibodies. Pharmacoepidemiology and Drug Safety.

[5] Kestenbaum B. Methods to Control for Confounding. In: Epidemiology and Biostatistics. New York: Springer: 2009. p. 101–11.

[6] (Rothman KJ, Greenland S, Lash TL, editors.)Modern Epidemiology. 530 Walnut Street, Philadelphia, PA 19106 USA: Lippincott Williams & Wilkins; 2008.

[7] Glynn RJ, Schneeweiss S, Sturmer T (2006). Indications for Propensity Scores and Review of Their Use in Pharmacoepidemiology. Basic Clin Pharmacol Toxicol.

[8] Rosenbaum PR, Rubin DB (1983). The central role of the propensity score in observational studies for causal effects. Biometrika.

[9] Austin PC (2011). A Tutorial and Case Study in Propensity Score Analysis: An Application to Estimating the Effect of In-Hospital Smoking Cessation Counseling on Mortality. Multivar Behav Res.

[10] Austin PC (2014). The use of propensity score methods with survival or time-to-event outcomes: reporting measures of effect similar to those used in randomized experiments. Stat Med.

[11] Lunceford JK, Davidian M (2004). Stratification and weighting via the propensity score in estimation of causal treatment effects: a comparative study. Stat Med.

[12] Austin PC, Grootendorst P, Normand S-LT, Anderson GM (2007). Conditioning on the propensity score can result in biased estimation of common measures of treatment effect: a Monte Carlo study. Stat Med.

[13] Austin PC (2008). The performance of different propensity-score methods for estimating relative risks. J Clin Epidemiol.

[14] Austin PC (2009). Some methods of propensity-score matching had superior performance to others: results of an empirical investigation and Monte Carlo simulations. Biom J Biom Z.

[15] Austin PC (2010). The performance of different propensity-score methods for estimating differences in proportions (risk differences or absolute risk reductions) in observational studies. Stat Med.

[16] Austin PC (2013). The performance of different propensity score methods for estimating marginal hazard ratios. Stat Med.

[17] Rosenbaum PR, Rubin DB (1984). Reducing Bias in Observational Studies Using Subclassification on the Propensity Score. J. Am Stat Assoc.

[18] Rubin DB, Thomas N (1996). Matching Using Estimated Propensity Scores: Relating Theory to Practice. Biometrics.

[19] Rosenbaum PR (1987). Model-Based Direct Adjustment. J Am Stat Assoc.

[20] Austin PC (2009). The relative ability of different propensity score methods to balance measured covariates between treated and untreated subjects in observational studies. Med Dec Mak An Int J Soc Med Dec Mak.

[21] Sturmer T, Joshi M, Glynn RJ, Avorn J, Rothman KJ, Schneeweiss S (2006). A review of the application of propensity score methods yielded increasing use, advantages in specific settings, but not substantially different estimates compared with conventional multivariable methods. J Clin Epidemiol.

[22] Pirracchio R, Resche-Rigon M, Chevret S (2012). Evaluation of the Propensity score methods for estimating marginal odds ratios in case of small sample size. BMC Med Res Methodol.

[23] Cepeda MS, Boston R, Farrar JT, Strom BL (2003). Comparison of Logistic Regression versus Propensity Score When the Number of Events Is Low and There Are Multiple Confounders. Am J Epidemiol.

[24] Patorno E, Glynn RJ, Hernández-Díaz S, Liu J, Schneeweiss S (2014). Studies with many covariates and few outcomes: selecting covariates and implementing propensity-score-based confounding adjustments. Epidemiol (Cambridge, Mass).

[25] Leyrat C, Caille A, Donner A, Giraudeau B. Propensity score methods for estimating relative risks in cluster randomized trials with low-incidence binary outcomes and selection bias. Stat Med. 2014. doi:10.1002/sim.6185. 10.1002/sim.6185 24771662

[26] Rassen JA, Schneeweiss S (2012). Newly marketed medications present unique challenges for nonrandomized comparative effectiveness analyses. J Comp Eff Res.

[27] Arbogast PG, Seeger JD, DEcIDE Methods Center Summary Variable Working Group. Summary Variables in Observational Research: Propensity Scores and Disease Risk Scores. Effective Health Care Program Research Report No. 33. (Prepared by DEcIDE Methods Center under Contract No. HHSA 290-2005-0016-I, Task Order 10.) AHRQ Publication No. 11(12)-EHC055-EF. Rockville, MD: Agency for Healthcare Research and Quality. May 2012. http://effectivehealthcare.ahrq.gov/reports/final.cfm.

[28] Velentgas P, Dreyer NA, Nourjah P, Smith SR, Torchia MM, (eds). Developing a Protocol for Observational Comparative Effectiveness Research: A User’s Guide. AHRQ Methods for Effective Health Care. Rockville (MD): Agency for Healthcare Research and Quality (US);2013. 23469377

[29] Arbogast PG, Ray WA. Performance of Disease Risk Scores, Propensity Scores, and Traditional Multivariable Outcome Regression in the Presence of Multiple Confounders. Am J Epidemiol. 2011;143. [doi:10.1093/aje/kwr143]. 10.1093/aje/kwr143 21749976

[30] Wyss R, Ellis AR, Brookhart MA, Jonsson Funk M, Girman CJ, Simpson RJ, Stürmer T (2015). Matching on the disease risk score in comparative effectiveness research of new treatments. Pharmacoepidemiol Drug Saf.

[31] Havercroft WG, Didelez V (2012). Simulating from marginal structural models with time-dependent confounding. Stat Med.

[32] Austin PC (2008). A critical appraisal of propensity-score matching in the medical literature between 1996 and 2003. Stat Med.

[33] Imbens G. Nonparametric Estimation of Average Treatment Effects under Exogeneity: A Review. Rev Econ Stat. 2004.

[34] Robins JM, Hernán MÁ, Brumback B (2000). Marginal Structural Models and Causal Inference in Epidemiology. Epidemiology.

[35] Cole SR, Hernán MA (2008). Constructing inverse probability weights for marginal structural models. Am J Epidemiol.

[36] Austin PC (2011). Optimal caliper widths for propensity-score matching when estimating differences in means and differences in proportions in observational studies. Pharm Stat.

[37] Burton A, Altman DG, Royston P, Holder RL (2011). The design of simulation studies in medical statistics. Stat Med.

[38] Eddelbuettel D, Francois R (2011). Rcpp: Seamless R and C++ Integration. J Stat Softw.

[39] Roussel R, Hadjadj S, Pasquet B, Wilson PW, Smith SC, Goto S, Tubach F, Marre M, Porath A, Krempf M, Bhatt DL, Steg PG (2013). Thiazolidinedione use is not associated with worse cardiovascular outcomes: a study in 28,332 high risk patients with diabetes in routine clinical practice: brief title: thiazolidinedione use and mortality. Int J Cardiol.

[40] Bhatt DL, Eagle KA, Ohman EM, Hirsch AT, Goto S, Mahoney EM, Wilson PWF, Alberts MJ, D’Agostino R, Liau C-S, Mas J-L, Röther J, Smith SC, Salette G, Contant CF, Massaro JM, Steg PG, REACH Registry Investigators (2010). Comparative determinants of 4-year cardiovascular event rates in stable outpatients at risk of or with atherothrombosis. JAMA.

[41] Steg PG, Bhatt DL, Wilson PWF, D’Agostino R, Ohman EM, Röther J, Liau C-S, Hirsch AT, Mas J-L, Ikeda Y, Pencina MJ, Goto S, REACH Registry Investigators (2007). One-year cardiovascular event rates in outpatients with atherothrombosis. JAMA.

[42] Ohman EM, Bhatt DL, Steg PG, Goto S, Hirsch AT, Liau C-S, Mas J-L, Richard A-J, Röther J, Wilson PWF, REACH Registry Investigators (2006). The REduction of Atherothrombosis for Continued Health (REACH) Registry: an international, prospective, observational investigation in subjects at risk for atherothrombotic events-study design. Am Heart J.

[43] Bhatt DL, Steg PG, Ohman EM, Hirsch AT, Ikeda Y, Mas J-L, Goto S, Liau C-S, Richard AJ, Röther J, Wilson PWF, REACH Registry Investigators (2006). International prevalence, recognition, and treatment of cardiovascular risk factors in outpatients with atherothrombosis. JAMA.

[44] Lavandeira A (2002). Orphan drugs: legal aspects, current situation. Haemophilia: The Official J World Fed Hemophilia.

[45] Austin PC, Schuster T. The performance of different propensity score methods for estimating absolute effects of treatments on survival outcomes: A simulation study. Stat Methods Med Res. 2014. 0962280213519716, doi:10.1177/0962280213519716. 10.1177/0962280213519716 PMC5051602 24463885

[46] Hansen BB (2008). The prognostic analogue of the propensity score. Biometrika.

[47] Glynn RJ, Gagne JJ, Schneeweiss S (2012). Role of disease risk scores in comparative effectiveness research with emerging therapies. Pharmacoepidemiol Drug Saf.

[48] Westreich D, Lessler J, Funk MJ (2010). Propensity score estimation: neural networks, support vector machines, decision trees (CART), and meta-classifiers as alternatives to logistic regression. J Clin Epidemiol.

[49] Vittinghoff E, McCulloch CE (2007). Relaxing the Rule of Ten Events per Variable in Logistic and Cox Regression. Am J Epidemiol.

[50] Peduzzi P, Concato J, Kemper E, Holford TR, Feinstein AR (1996). A simulation study of the number of events per variable in logistic regression analysis. J Clin Epidemiol.

[51] Greenland S (1987). Interpretation and Choice of Effect Measures in Epidemiologic Analyses. Am J Epidemiol.

[52] Gail MH, Wieand S, Piantadosi S (1984). Biased Estimates of Treatment Effect in Randomized Experiments with Nonlinear Regressions and Omitted Covariates. Biometrika.

[53] Xiao Y, Abrahamowicz M, Moodie EEM (2010). Accuracy of Conventional and Marginal Structural Cox Model Estimators: A Simulation Study. Int J Biostat.

