# Supplementary material for: On the use of propensity scores in case of rare exposure
Source: BMC Med Res Methodol. 2016 Mar 31;16:38. doi: 10.1186/s12874-016-0135-1 (PMC4815252; doi:10.1186/s12874-016-0135-1)
Supplement: Additional file 1 — Data-generation process and simulated scenarios. (DOCX 119 kb) [file 12874_2016_135_MOESM1_ESM.docx]

# Definitions

- E: indicator variable denoting exposure status (E = 1 for exposed subjects, E = 0 otherwise)
- Y: indicator variable of the event of interest (Y = 1 if subject has experimented the event, Y = 0 otherwise)
- t: observed follow-up time.
- B, B’, C and C’: two baseline covariates (confounding factors)
- U: unmeasured latent general health baseline variable

# Relationships between covariates

# Simulation parameters

- the prevalence of exposure: p ∈ {1%,2%,5%,10%};
- the number of confounding factors: one binary (B) and one continuous (C), or two binaries (B and B’) and two continuous (C and C’)
- the strength of the correlation between covariates B and C, and between covariates B’ and C’: σ_B,C =_ σ_B’,C’_ ∈ {0,0.1,0.3,0.5} (no, weak, moderate, or strong correlation);
- the strength of the association between covariates and U: σ_U,B_ = σ_U,C_ = σ_U,B’_ = σ_U,C’_ ∈ {0,0.1,0.3,0.5} (no, weak, moderate, or strong association);
- the strength of the association between covariates and exposure allocation: exp(δ_B_) = exp(δ_C_) = exp(δ_B’_) = exp(δ_C’_) ∈ {1,1.2,1.5,2} (no, weak, moderate, or strong association);
- the strength of the marginal association between exposure and outcome: HR = exp(γ) ∈ {1,1.2,1.5,2} (no, weak, moderate, or strong association);
- the censoring rate: r_c_ ∈ {20%,50%,80%}.

# Data generating process

1) randomly generate of five normally distributed covariates (sample size N = 10000):

X = [X_B_, X_C_, X_B’_, X_C’_, X_U_] ∼ N(0,Σ) with correlation matrix Σ = $\left[ \begin{matrix} 1 & \sigma_{B,C} & 0 & 0 & \sigma_{U,B} \\ \sigma_{B,C} & 1 & 0 & 0 & \sigma_{U,C} \\ 0 & 0 & 1 & \sigma_{B',C'} & \sigma_{U,B'} \\ 0 & 0 & \sigma_{B',C'} & 1 & \sigma_{U,C'} \\ \sigma_{U,B} & \sigma_{U,C} & \sigma_{U,B'} & \sigma_{U,C'} & 1 \end{matrix} \right]$

2) transform each variable as follows:

- B = 1 if X_B_ > 0, B = 0 if X_B_ ≤ 0

- B’ = 1 if X_B’_ > 0, B’ = 0 if X_B’_ ≤ 0

- C = X_C_,

- C’ = X_C’_,

- U = P(X_U_ < x)

3) draw the exposure allocation E from a Bernoulli distribution E ∼ B(p_z_), where :

- p_z_ = logit^−1^ (δ_0_ + δ_B_ B + δ_C_ C) when scenario implies variables B and C only, or

- p_z_ = logit^−1^ (δ_0_ + δ_B_ B + δ_C_ C + δ_B’_ B + δ_C’_ C) when scenario implies variables B, B’, C and C’,

δ_0_ is chosen using an iterative process so that exposure prevalence in the simulated sample is fixed at a desired proportion p.

4) generate the event time T with exponential distribution as follows:

T =−Log(U)/(λexp(γ E)) with λ = 0.1

5) generate the censoring time T_c_ drawn from a uniform distribution U(0,c), where c is chosen using an iterative process to achieve a desired censoring rate r_c_ in the simulated sample.

6) obtain the observed time-to-event outcome with the following decision rule:

- Y = 1, t = T if T <= T_c_

- Y = 0, t = T_c_ if T > T_c_

# Reported scenarios

All simulation parameters were crossed using a factorial design, resulting in 6144 different scenarios.

For the sake of concision, only a limited number of scenarios were reported in the ‘Results’ section. We first chose a reference configuration, and then reported the effects of change of each of the simulation parameters on the results.

The above table lists all the reported scenarios (**the reference configuration is highlighted in bold font** on each line).

|  | Exposure prevalence | Confounding factors B, B’, C and C’ | Correlation between covariates B/C and B’/C’ | Correlation between covariates and U | Association between covariates and exposure allocation | Marginal association between exposure and outcome (HR) | Censoring rate |
| --- | --- | --- | --- | --- | --- | --- | --- |
| **Reference configuration** | **5%** | **B and C only** | **No** | **Moderate** | | **1** | **50%** |
| Scenarios with a varying prevalence | 1%-2%-**5%**-10% | **B and C only** | **No** | **Moderate** | | **1** | **50%** |
| Scenarios with a varying HR | **5%** | **B and C only** | **No** | **Moderate** | | **1**-1.2-1.5-2 | **50%** |
| Scenarios with a varying strength of confounding | **5%** | **B and C only** | **No** | No-Weak-**Moderate**-Strong | | **1** | **50%** |
| Scenario with a varying number of confounding factors | **5%** | **B and C only**-  B, B’, C and C’ | **No** | **Moderate** | | **1** | **50%** |
| Scenario with a varying censoring rate | **5%** | **B and C only** | **No** | **Moderate** | | **1** | 20%-**50%**-80% |
| Scenario with a varying correlation between covariates | **5%** | **B and C only** | **No**-Weak-Moderate-Strong | **Moderate** | | **1** | **50%** |
